# Therapy preference of 131 parents confronted with a pediatric femoral fracture

**DOI:** 10.3389/fped.2022.949019

**Published:** 2022-08-15

**Authors:** Christoph Arneitz, Istvan Szilagyi, Bianca Lehner, Bernhard Kienesberger, Paolo Gasparella, Christoph Castellani, Georg Singer, Holger Till

**Affiliations:** ^1^Department of Paediatric and Adolescent Surgery, Medical University of Graz, Graz, Austria; ^2^Department of Paediatric and Adolescent Surgery, Clinical Center Klagenfurt, Klagenfurt, Austria

**Keywords:** pediatric femoral fracture, over-head extension, elastic stable intramedullary nailing, personality traits, Freiburg Personality Inventory

## Abstract

**Background:**

The management of femoral fractures in children between 3 and 5 years of age is still vividly debated. Therefore, we aimed to assess the basic attitude of parents if confronted with a hypothetical femoral fracture of their toddler.

**Materials and methods:**

Parents of children aged between 12 and 36 months were asked for their preference after receiving detailed information on conservative and surgical treatment of femoral shaft fractures. Furthermore, we obtained information regarding the parents’ gender, marital status, medical background, highest level of education and profession in a leading or non-leading position and if any of their children already had undergone any operations. The Freiburg Personality Inventory (FPI-R) questionnaire was used to assess parents’ personality traits.

**Results:**

In total, 131 participants were included in this study. The vast majority (*n* = 116, 88.5%) preferred surgical treatment. The most frequently mentioned reasons for this decision were lack of acceptance, followed by faster reconvalescence, shorter hospital stay, less deformity or growth disorders and less stress on the child. The only reason stated against surgical treatment was the need of general anesthesia. A significantly higher rate of conservative procedures was noticed in self-employed participants and stress was found to significantly influence the treatment decision of the parents toward conservative treatment.

**Conclusion:**

The majority of parents confronted with a hypothetical femoral fracture of their child questioned in this study opted for a surgical approach with elastic stable intramedullary nailing (ESIN). This corresponds with trends toward surgery in these cases in major trauma centers in Europe.

## Introduction

Femoral shaft fractures represent 1.3% of all childhood fractures with an overall annual incidence of 15–25 per 100,000 children (1–3). There are two known peaks of incidence, one in early childhood and another in mid-adolescence (4). One third of femoral fractures occur in children below 5 years of age (5). Femoral fractures in early childhood are a domain of conservative fracture treatment. Consequently, the majority of published guidelines advocate either over-head extension (OHE) followed by application of a single-leg spica cast (SC) or immediate application of the cast (4–7). The “*traction and cast*” approach usually requires inpatient treatment for approximately 8–14 days until sufficient callus provides enough stability to apply a SC for another 1–3 weeks; therefore, patients remain immobilized for 3–5 weeks (4, 8, 9). Immediate application of SC is often not possible without general anesthesia due to initial malalignment and in addition, the soft tissue to bone relation and muscular traction may still promote secondary displacement. Moreover, leg length discrepancy, malunion and skin breakdown are known complications (10, 11).

Recently, however, surgical treatment using elastic stable intramedullary nailing (ESIN) has gained increased acceptance for fracture treatment even in toddlers (11). Especially in large trauma centers, femoral fractures in early childhood are increasingly treated surgically (4, 5). Only a few studies have directly compared conservative and surgical management of pediatric femoral fractures in preschool-aged children (12–15). Authors using ESIN in children younger than 5 years of age mention better fracture reduction, a quicker discharge (the mean inpatient duration is 6.4 days), and a faster weight-bearing after an average of 14.1 days without an increase of morbidity as major advantages of this approach (11, 13, 14, 16, 17). However, ESIN requires a second operation in general anesthesia after approximately 6 months for implant removal and is associated with potential but rare complications such as displacement with shortening or varus deviation (reported in 3.3% of the cases) and minor restrictions without the need for revision such as hematomas, hypertrophic scarring, and temporary functional impairments in up to 25% of patients treated with ESIN (18).

The general attitude of parents concerning conservative or surgical treatment is an important factor since positive parental support is essential for treatment of pediatric patients (19, 20). In preschool-aged children with femoral fractures, both treatment methods are possible without clear medical evidence of benefits for either approach. At present, however, the general attitude of parents toward either option remains unelucidated. Therefore, it was the aim of this study to assess the basic attitude of parents toward surgical or conservative management if confronted with a hypothetical femoral fracture of their toddler. Furthermore, we investigated whether and which parents’ personality traits impact their treatment choice.

## Materials and methods

**FIGURE 1:**
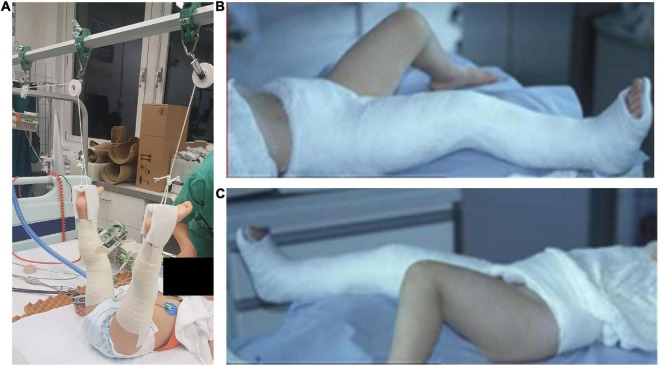
Conservative treatment: The child is placed in a supine position and the legs are extended overhead under traction for 8–14 days **(A)** followed by a single-leg spica for another 1–3 weeks **(B,C)**.

After approval by the institutional review board (EK 32-214 ex 19/20) anonymized information sheets giving detailed information on conservative and surgical treatment of femoral shaft fractures were distributed to 27 public day nurseries (crèches) in Graz caring for children aged from 12 to 36 months and among the personal environment of students and colleagues. Informed consent was provided to all participants and the information sheet contained photographs and information regarding the following treatment options:

For conservative treatment, the child is placed in a supine position and the legs are extended overhead under tension for 10 days (Figure 1). After 10 days, an X-ray is performed. If the bone is in a good position and bone healing is already visible, a long-leg SC is applied for further 2 weeks. The hospital stay is 10 days, weight-bearing is possible after 3–4 weeks and misalignment and growth disorders can occur. Growth disorders were mainly considered as growth stimulation disorders with consecutive leg-length discrepancy, which are known to usually remodel in the further course (4).

**FIGURE 2:**
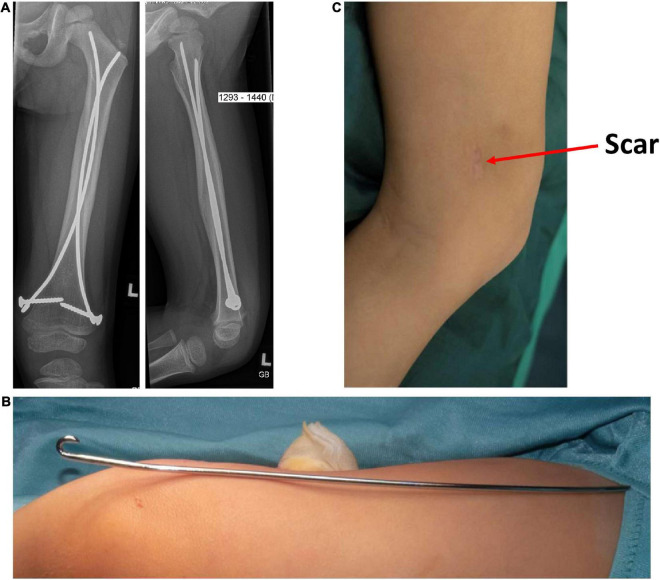
Surgical treatment: Postoperative radiographs are shown to the parents including pictures of the elastic, stable intramedullary nail **(A,B)** and the scar formation marked with an arrow **(C)**.

In case of surgical treatment, ESIN is performed under general anesthesia. In case of oblique fractures, interlocking screws are used to prevent migration of the nails (Figure 2). Postoperatively, the bone is load-resistant even in oblique fractures. The length of hospital stay ranges from 5 to 7 days, the bone usually heals in an axially aligned position and weight-bearing is usually possible immediately after the operation. However, an additional operation under general anesthesia is required to remove the implants after approximately 6 months.

After receiving the information, participants were asked for their preference and to briefly justify their decision. Furthermore, we obtained information regarding the parents’ gender, marital status, medical background, highest level of education and profession in a leading or non-leading position and if any of their children already had undergone an operation.

The Freiburg Personality Inventory (FPI-R) questionnaire was used to assess parents’ personality traits. The questionnaire contains 138 items assessing levels of the following personality traits: life satisfaction, social orientation, performance orientation, inhibition, excitability, aggressiveness, stress, physical complaints, health concerns, openness, extraversion, and emotionality. The evaluation of the completed questionnaires was carried out with the software “FPI-R” Ver. 3.2 (21).

Data was entered into an Excel 2019^®^ [Microsoft Corporation. Microsoft Excel (Internet). 2018, United States] spreadsheet and then transferred to SPSS Statistics 21^©^ (IBM Corp. Released 2012. IBM SPSS Statistics for Windows, Version 21.0. Armonk, NY: IBM Corp.) for statistical analysis. A Kolmogorov Smirnov test was applied to assess normal distribution and Levene’s Test to determine the homogeneity of variances. In case of normal distribution, data are displayed as mean and standard deviation and statistical group comparison was performed using a 2-sided, unpaired *t*−test. In case of absent normal distribution, data are displayed as median and interquartile range (IQR). In this case and for ordinal data, a Mann-Whitney-*U*-test was used for group comparison. Categorical data were compared with the Chi-squared test. Explorative statistical significance was defined as *p* < 0.05.

## Results

A total of 131 persons agreed to participate in this study, none had to be excluded. The majority of respondents participating in this study was female (*n* = 103; 78.6%); 73 (55.7%) reported having more than one child; 91 (69.5%) mentioned previous surgeries of parents (*n* = 60; 45.8%), their children (*n* = 9; 6.9%) or both (*n* = 22; 16.8%). Considering the marital status, the results revealed that 94 (71.7%) were married, 36 (27.5%) single, and in 1 (0.8%) divorced. Finally, 57 (43.5%) participants had a medical background.

The vast majority (*n* = 116, 88.5%) preferred surgical treatment. The reasons most frequently mentioned were lack of acceptance, followed by faster reconvalescence, shorter hospital stay, lower probability of deformity or growth disorders and less stress on the child (Table 1). Furthermore, psychological stress and consequences of the OHE and the child’s urge to move were mentioned. The only reason against surgical treatment given by parents was the need of general anesthesia. The unreasonableness of the fixation and the faster reconvalescence correlated significantly with surgical treatment while the need of anesthesia significantly correlated with conservative therapy (Table 1).

**TABLE 1:** Correlation of reasons mentioned and treatment decision.

	*n*	*X* ^2^	*P*
**Surgical treatment**			
Lack of acceptance	67	*X*^2^ (1, *N* = 131) = 17.734	***p* = 0.001**
Faster reconvalescence	42	*X*^2^ (1, *N* = 131) = 7.994	***p* = 0.002**
Shortening the hospital stay	18	*X*^2^ (1, *N* = 131) = 2.698	*p* = 0.095
Lower probability of deformity or growth disorders	18	*X*^2^ (1, *N* = 131) = 2.698	*p* = 0.095
Less stress on the child	18	*X*^2^ (1, *N* = 131) = 2.698	*p* = 0.095
Psychological stress of the OHE	17	*X*^2^ (1, *N* = 131) = 2.526	*p* = 0.109
Child’s urge to move	15	*X*^2^ (1, *N* = 131) = 2.190	*p* = 0.144
**Conservative treatment**			
Need of anesthesia	14	*X*^2^ (1, *N* = 131) = 2.698	***p* = 0.001**

A Chi-squared-test was applied for comparison. Bold values denote statistical significance. OHE, overhead extension.

Children of parents opting for surgical treatment (*n* = 116) had a mean age of 24.6 ± 8.3 months. There was no significant difference to children of parents choosing conservative management (*n* = 15, 28.1 ± 7.9 months; *p* = 0.115, *t*-test). The mean age of the parents was 35.7 ± 4.1 years in the conservative and 34.8 ± 4.3 years in the surgical group (*p* = 0.435, *t*-test).

Parents’ gender, marital status, and prevalence of previous surgeries, medical background, highest level of education, and profession in a leading or non-leading position were not significantly different between the surgical and conservative group (Table 2). A significantly higher rate of conservative procedures was noticed in self-employed participants (*p* = 0.041, Chi squared test).

**TABLE 2:** Treatment decision based on social criteria.

		Treatment (*n*)		*P*-value
		Conservative	Surgical	
Gender (parents)	Male	4	24	0.595
	Female	11	92	
Family status	Only child	8	50	0.453
	More children	7	66	
Marital status	Single/divorced	5	32	0.642
	Married	10	84	
Medical background	No	9	65	0.771
	Yes	6	51	
Previous surgeries	None	6	34	0.398
	Child and/or parent	9	82	
Highest education	University	5	36	0.571
	Secondary school	6	62	
Profession	Employed	10	100	**0.041**
	Self-employed	5	15	
Profession 2	No leading position	12	101	0.398
	Leading position	3	14	

A Chi-Squared test was applied for group comparison. Bold values denote statistical significance.

Statistical analysis of the FPI-R showed no significant differences in life satisfaction, social orientation, performance orientation, inhibition, excitability, aggressiveness, physical complaints, health concerns, openness, extraversion or emotionality (Table 3). However, stress was found to significantly influence the treatment decision of the parents (*p* = 0.014, Mann-Whitney-*U*-test).

**TABLE 3:** Treatment decision based on FPI-R criteria; ordinal data are displayed as median and interquartile range (IQR).

FPI-R	Treatment		*P*-value
	Conservative	Surgical	
Life satisfaction	6 ± 3	6 ± 2	0.283
Social orientation	7 ± 3	6 ± 2	0.174
Performance orientation	5 ± 2	5 ± 2	0.791
Inhibition	5 ± 2	5 ± 2	0.714
Excitability	5 ± 3	5 ± 3	0.348
Aggressiveness	3 ± 3	3 ± 2	0.956
Stress	5 ± 2	4 ± 2	**0.014**
Physical complaints	5 ± 3	4 ± 2	0.364
Health organ	5 ± 2	5 ± 2	0.643
Openness	5 ± 3	5 ± 2	0.176
extraversion	5 ± 2	5 ± 3	0.491
Emotional instability	5 ± 3	4 ± 2	0.083

A Mann-Whitney-U-test was used for group comparison. Bold values denote statistical significance.

## Discussion

This questionnaire study has examined the parents’ therapy preferences for femoral shaft fractures in toddlers combined with an assessment of the parents’ personality traits. The main findings of the present study were that most parents preferred the surgical approach; stress and self-employment were found to be significantly linked to the preference for a conservative therapy regimen.

Treatment options of pediatric femoral shaft fractures have undergone fundamental changes during the last years. A surgical approach with ESIN has the advantages of rapid recovery, shorter hospital stay, less muscle atrophy and low possibility of fracture malunion compared to conservative therapy (22). Consequently, surgical treatment of femoral shaft fractures in children younger than 3 years is already a clinical standard in the majority of trauma surgery and pediatric surgery departments in Germany (5).

Scientific research focusing on this specific age group are rare. Only a few studies have directly compared conservative and surgical management of femoral fractures in toddlers and found favorable outcome (better fracture reduction, a quicker discharge and a faster weight-bearing) following ESIN also in preschool-aged children (1, 11, 12, 14, 16). However, ESIN requires a second operation in general anesthesia after approximately 6 months for implant removal and is associated with potential but rare complications such as displacement with shortening or varus deviation (reported in 3.3% of the cases) and minor restrictions without the need for revision such as hematomas, hypertrophic scarring and temporary functional impairments in up to 25% of patients treated with ESIN (18).

A recently published meta-analysis of treatment of closed femoral shaft fractures in children aged 2–10 years including 21 studies with 1,675 patients found significantly lower rates of malunion, lower means of angulation and shortening and earlier achievement of rehabilitation in children treated with ESIN compared with both immediate spica casting and traction and subsequent casting (11). Moreover, also a subgroup analysis of children aged between 2 and 6 years showed superior radiological outcomes and faster reconvalescence in the ESIN group (11). Shorter hospital stay, early mobilization and weight bearing were mentioned as additional benefits of ESIN in the literature (16, 23, 24).

However, in pediatric femoral fractures, still both treatment methods (surgical or conservative) are reasonable and no consensus on treatment could be reached. Medical care of young patients implies shared decision-making with their parents regarding available treatment options. In this decision-making process, detailed information must be provided including advantages and disadvantages of possible procedures or additional therapeutic options. If there are more than one available treatment options, parents must weigh the factors above in order to decide which treatment is most appropriate for their child. Subsequently, parents may experience a range of affective distress, including anxiety, depression, and constant worry. The severity with which this distress is expressed may be related to various psychosocial factors including personality traits of the parents, child’s age and family stress resilience (19, 20).

A major determinant of parents’ decision making when considering either surgical or conservative treatment appears to be stress. Acute stress of parents has been shown to be related to the type of surgical treatment their children receive (20). In our study, parents who report high levels of stress as a personality construct and who experience frequent time pressure, high levels of tension or excessive workload, amongst others, are likely to choose different treatment modalities for their children compared to parents with lower levels of this personality construct.

Since positive parental support is essential in the treatment of pediatric patients, their support regarding the choice of treatment plays an important role (19, 20). At present, however, the general attitude of parents toward conservative or surgical treatment of femoral fractures remains unelucidated. Given the rare number of pediatric femoral fractures and different treatment strategies in pediatric trauma centers, representative surveys of parents’ treatment choices would require a huge effort. Additionally, parents with injured children suffer from acute stress (20). Therefore, additional questions regarding their background as required for a comparative study might be inappropriate and a decision has to be made under time pressure and stress. At present, only two studies had compared the parent satisfaction after different treatment regimens of femoral fractures and found a significant higher satisfaction in the ESIN group (13, 24). Buechsenschuetz et al. compared 16 conservatively treated patients with 27 patients treated with ESIN and found that 93% of the ESIN group would choose the treatment again compared to only 6% in patient treated with traction and subsequent casting (*p* < 0.001) (13). However, both studies included mostly children older than 5 years which might lead to higher dissatisfaction in the conservative group. In this regard only one study has examined the satisfaction of parents with OHE in children below the age of 4 years, but did not compare it with other treatment methods (9). The authors found a high rate of parent dissatisfaction; 26.7% of parents would not decide for this treatment again and the OHE appeared to be stressful for many parents and children (9). Furthermore, 70% of the parents noticed “behavioral problems” in their child during the time of fixation and 63% mentioned therapy-specific problems (9). These results confirm our findings that the unreasonableness of the fixation was the most often mentioned reason against conservative treatment and correlated significantly with the decision for a surgical approach.

In our investigation, stress was found to significantly influence the treatment decision of the parents. Parents with higher levels of stress tended toward conservative treatment. This is in contrast to recent findings, suggesting that stress modulates the individual propensity to engage in risk-taking (25). However, gender dependent differences were reported when confronted with risky decisions, with males taking more risk under stress (26). In this regard, the high rate of females (78.6%) in the present study could be a possible reason for the preference for conservative treatment in stressed participants.

Regarding employment, one might suspect that entrepreneurs would choose a treatment method granting early return to their business. Consequently, a tendency toward a surgical procedure, offering the benefits of short hospital stay and faster reconvalescence could be expected. Interestingly, we found a significantly higher rate of choices for conservative treatment in self-employed participants. The underlying reason for this preference remains to be elucidated. However, the case numbers of self-employed participants in this study are small and larger series are warranted to confirm our findings.

Limitations of the present study are the study design with a questionnaire study in parents confronted with a hypothetical femoral fracture of their child. OHE is often well tolerated by the child as well as the care-givers and better accepted as initially expected. However, the present study design can also be seen as an advantage since the decision was made carefully considered without the pressure for a quick decision in the emergency room next to the injured child.

## Conclusion

In conclusion, surgical treatment with ESIN appears to be the method of choice for parents of toddlers confronted with a hypothetical femoral fracture. These results correspond with international trends in the care of femoral fractures in children below 5 years of age and should be considered for future guidelines.

## Data availability statement

The raw data supporting the conclusions of this article will be made available by the authors, without undue reservation.

## Ethics statement

The study has been approved by the Ethics Committee of the Medical University of Graz (EK 32-214 ex 19/20).

## Author contributions

CA: study conception and design, data acquisition, statistical analysis, data interpretation, and manuscript preparation. IS, GS, and CC: study design, statistical analysis, and manuscript preparation. BL: data acquisition and manuscript preparation. BK: study design, data acquisition, and manuscript preparation. PG and HT: study design, manuscript preparation, and critical revision. All authors contributed to the article and approved the submitted version.
